# Evaluation of cardiac function and systolic dyssynchrony of fetuses exposed to maternal autoimmune diseases using speckle tracking echocardiography

**DOI:** 10.1007/s10067-021-05723-6

**Published:** 2021-04-04

**Authors:** ShaSha Duan, Si Ha, ShuJuan Li, YaXi Wang, YiLu Shi, HaiYue Zhao, Lu Zhang, XiaoShan Zhang, Yong Wang

**Affiliations:** 1 Department of Ultrasound, The Affiliated Hospital of Inner Mongolia Medical University, Hohhot, 010050 China; 2 Department of Ultrasound, Second Affiliated Hospital of Harbin Medical University, Harbin, China; 3 Department of Emergency, The Affiliated Hospital of Inner Mongolia Medical University, Hohhot, China; 4 Department of Rheumatology, The Affiliated Hospital of Inner Mongolia Medical University, Hohhot, 010050 China

**Keywords:** Antibodies, Anti-SSA, Anti-SSB, Echocardiography, Systolic dyssynchrony

## Abstract

**Objectives:**

To compare cardiac function and systolic dyssynchrony of fetuses not exposed to and those exposed to maternal autoimmune antibodies using two-dimensional speckle tracking echocardiography (2DSTE).

**Methods:**

An observational study of 52 fetuses, 18 from mothers with autoimmune antibodies (anti-SSA/Ro60, anti-Ro52 or/and anti-SSB/La) and 34 from healthy mothers without antibodies, was conducted. Maternal baseline characteristics, fetoplacental Doppler parameters, and conventional echocardiographic data were prospectively collected. Systolic global and regional longitudinal strain of left and right ventricle (LV and RV) and the time to peak strain of regional myocardium were measured using 2DSTE. We also calculated the differences in time to peak strain between the LV free wall and RV free wall (two-chamber dyssynchrony, 2C-DYS) and the LV dyssynchrony between the septum and LV free wall (one-chamber dyssynchrony, 1C-DYS).

**Results:**

There were no significant differences in conventional systolic and diastolic functional parameters for the LV and RV. No effect modification was demonstrated in a myocardial deformation analysis. However, 1C-DYS was significantly more prolonged in the maternal autoimmune disease group (19.50 [8.00 to 29.25] vs. 28.50 [13.50 to 39.25], *P* = 0.042).

**Conclusions:**

LV systolic mechanical dyssynchrony in fetuses of mothers with autoimmune antibodies suggests in-utero subclinical damage of the cardiac conduction system.

**Table:** 

Key points *• The left ventricular systolic dyssynchrony was significantly more prolonged in the maternal autoimmune disease (AD) fetuses.* *• Subclinical damage to the left ventricular conduction system of the fetal heart in maternal AD was observed.* *• Systolic and diastolic functional of the left and right ventricle were preserved in fetuses exposed to maternal autoimmune disease.*

## Introduction

Autoimmune diseases (AD), including rheumatoid arthritis, systemic lupus erythematosus, and Sjogren’s syndrome, are more common in women of childbearing age [1]. Intra-uterine exposure of the fetus to maternal autoantibodies, especially if the mother has tested positive for the autoantibodies (anti-SSA/Ro or/and anti-SSB /La), increases the risk of damage to the fetal conductive system and cardiac function. Maternal IgG homologous autoantibodies can be transferred to the fetus via the placenta resulting in autoantibody-associated congenital heart block (CHB) and heart failure [2]. Although autoantibody-associated CHB may initially present as a first- or second-degree heart block, most patients will have a third-degree (complete) heart block. In fetuses of women with AD, the progression to CHB and cardiomyopathy is not sequential and could happen within a week of normal rhythm without preceding first degree block. A third-degree heart block is potentially lethal, and most fetuses require permanent pacemaker implantation after birth [3].

Fetal echocardiography remains the mainstay for cardiac evaluation and can assess cardiac structure, heart rhythm, anatomic abnormalities, ventricular function, and valvular function [4]. Conventional M-mode echocardiography and pulsed-wave Doppler techniques are used to diagnose atrioventricular block in fetuses of women with AD [5]. However, in the early stages of the disease, alterations in fetal cardiac function and dyssynchrony might be subtle in fetuses with normal heart rhythm and are difficult to quantify using conventional imaging.

Two-dimensional speckle tracking echocardiography (2DSTE) is a recent technology that tracks speckle patterns in the myocardium throughout the cardiac cycle. By calculating the myocardial deformation parameters (strain and time to peak strain), this technology provides a more accurate measure of cardiac function and dyssynchrony in fetuses, children, and adults, in normal and specific disease conditions than previous parameters [6–11].

To the best of our knowledge, cardiac function and systolic dyssynchrony of fetuses have not been well characterized in fetuses exposed to maternal autoantibodies. Therefore, the purpose of this study was to evaluate cardiac function and systolic mechanical dyssynchrony in fetuses of mothers with AD compared to fetuses of healthy mothers, exploring the potential role of deformation analysis and ventricular dyssynchrony using 2DSTE. We assumed that fetuses exposed to maternal autoimmune antibodies suffer ventricular dysfunction and dyssynchrony.

## Materials and methods

### Study population

An observational study was conducted at our hospital between July 2018 and November 2020. The research protocol was approved by the hospital ethics committee, NO. WZ (2020032). Informed consent was obtained from all participants. Fifty-two singleton pregnancies between 20 and 26 weeks of gestational age were enrolled in our study.

### Maternal autoantibody assay

The anti-SSA/Ro60, anti-Ro52, and anti-SSB /La antibody tests were performed twice, before pregnancy and at the second trimester respectively. The results of the two tests were consistent. The EUROLINE ANA Profile (IgG) (EUROIMMUN medical diagnosis Co., Ltd., Lubeck, Germany) was adopted, and the operation was carried out strictly in accordance with the reagent instructions. After completion of the tests, the results were categorized into four grades based on the depth of the film strip: −; ±; +, ++; and +++. We divided the pregnant women into two groups according to the test results, which were analyzed by two senior physicians. Moreover, the diagnosis was double-blind. Fetuses of 18 of these women (with [AD] with antibodies [anti-SSA/Ro60, anti-Ro52 or/and anti-SSB /La]) positive (+, ++, or +++) were randomly selected as the AD group. They were treated regularly with hydroxychloroquine (HCQ) before and during pregnancy, and some also treated with methylprednisolone. The control group fetuses were 34 randomly selected and matched with the cases by gestational age. All women in the control group were healthy with AD antibodies negative (−). Women in both groups had no history of smoking, alcohol abuse, hypertension, diabetes, or heart, liver, or other chronic diseases.

### Ultrasound protocol

The ultrasound examination was performed within 48 h following the antibody tested in the second trimester. Detailed fetal echocardiography was performed by an experienced cardiologist, according to the guidelines [12, 13]. Placental Doppler parameters were measured. All fetuses had a normal heart rhythm. Monochorionic twins and fetuses with cardiac anatomical anomaly, severe valve regurgitation, arrhythmias (atrial flutter, atrial fibrillation, and tachycardia), and poor echocardiographic image quality were excluded from the study. Maternal characteristics, including blood pressure, age, weight, and height, were collected during the ultrasound examination.

All echocardiographic parameters were acquired using a Vivid E95 ultrasound scanner (GE Vingmed Ultrasound AS, Horten, Norway) equipped with a S5–1 and a C1–6 transducer. The 2D cine loops were stored at a high frame rate (134 ~ 200 frames/s). Pulsed Doppler parameters were measured with the insonation angle <20. The analysis of deformations and dyssynchrony was performed offline using EchoPAC version 203 (GE Vingmed Ultrasound AS).

### Fetoplacental Doppler

We used the formula by Hadlock et al. [14] to estimate the fetal weight. Standard Doppler was used to measure the pulsatility index (PI) of the ductus venosus, umbilical artery, and middle cerebral artery. The cerebroplacental ratio was determined by dividing the PI of the middle cerebral artery by that of the umbilical artery [15].

### Fetal echocardiography

Cardiac morphometric and functional assessment by both conventional echocardiography and 2DSTE were performed. The morphometric measurement has been previously described [16]. Cardiac morphometry, including cardiothoracic ratio, ventricular sphericity index, left and right atrial areas, left ventricular shortening fraction, and mitral and tricuspid annular plane systolic excursion, were measured or calculated. The diastolic function included tricuspid and mitral early (E) and late (A) diastolic filling ratios (E/A ratio). The systolic function, including modified left and right myocardial performance index, was also assessed by tissue Doppler imaging.

A mechanical PR interval of the fetal heart was obtained by pulsed Doppler as described previously [17]. Left and right myocardial deformation and dyssynchrony parameters were analyzed using 2DSTE. A 2D video clip of a four-chamber view of the fetal heart was acquired for offline analysis. At least three loops from the four-chamber view were stored.

### Analysis of echocardiographic images

**Fig. 1:**
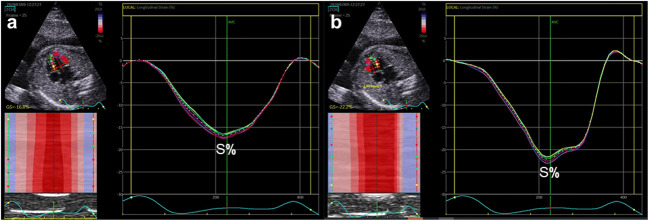
Global longitudinal strain analysis of left ventricle and right ventricle from the apical four-chamber view, with a graphic representation of average strain curves displayed as function of time for each of six segments. **a** Left ventricle. **b R**ight ventricle. S%, peak systolic strain

**Fig. 2:**
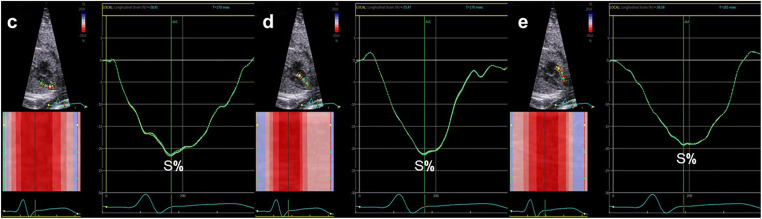
regional longitudinal strain analysis of left ventricle and right ventricle from the basal four-chamber view, with a graphic representation of average strain curves displayed as function of time for each of three segments. **c** LV free wall. **d** Interventricular septum. **e** RV free wall. S%, peak systolic strain

The 2D grayscale video clips of basal or apical four-chamber views were chosen. Because of the inability to record a fetal electrocardiograph, the mitral valve was followed to identify the beginning and end of one cardiac cycle, as previously described [6]. One cardiac cycle was stored for analysis. Global longitudinal strain (GLS) was calculated [18] (Fig. 1). The regional longitudinal strain and the time to peak strain of LV free wall (LVFW), RV free wall (RVFW), and interventricular septum (IVS) were analyzed from the same clip (Fig. 2). The ventricular endocardial borders were automatically recognized and could be manually adjusted if necessary. The LV included the IVS and LVFW, and the RV included the IVS and RVFW. LV systolic dyssynchrony and biventricular systolic dyssynchrony were calculated. In addition, we defined the standard deviations of the time to peak strain between the LVFW and RVFW as two-chamber dyssynchrony(2C-DYS) and within the LV between the IVS and the LVFW as one-chamber dyssynchrony(1C-DYS). All analyses were repeated with three different heart cycles.

### Sample size calculation

The sample size was determined by using the results from our preliminary experiment, in which the 1C-DYS was our primary outcome. We use it for the sample size calculation. The medians of the maternal AD group and the control group were 27.50 and 15.50 respectively, and the standard deviations (SDs) of the maternal AD group and the control group were 17.07 and 12.86, respectively. A two-tailed test was required with an alpha of 0.05, an statistical power of 85%, and the ratio between AD and control is 0.5. The sample size was calculated as the maternal AD group was 17, and 34 for the control group. Since this was a cross-sectional observational study, we determined a sample size of 18 in the maternal AD group and 34 in the control group.

### Statistical analyses

Categorical data were described as counts and proportions, and continuous data were expressed as mean ± SD and the median and interquartile range for skewed distributions. Normal distribution of the continuous data was determined by the Shapiro-Wilk test. Continuous variables were compared with an independent *t* test or Mann-Whitney *U* test, if appropriate. The chi-squared test or Fisher’s exact probability test was used for comparison of the classified data if applicable. For 2DSTE measurement, data from 10 random individuals were selected for inter- and intra-observer agreements and analyzed using the intra-class correlation coefficient (ICC) and Bland-Altman plots. We considered an ICC > 0.80 as excellent, 0.60 ≤ ICC ≤ 0.80 as good, 0.40 ≤ ICC ≤ 0.60 as moderate, and ICC < 0.40 as poor. All statistical analyses were performed using SPSS software (version 25.0, IBM Corp., Armonk, NY, USA). *P* < 0.05 was considered statistically significant.

## Results

### Baseline maternal and fetal characteristics

**Fig. 3:**
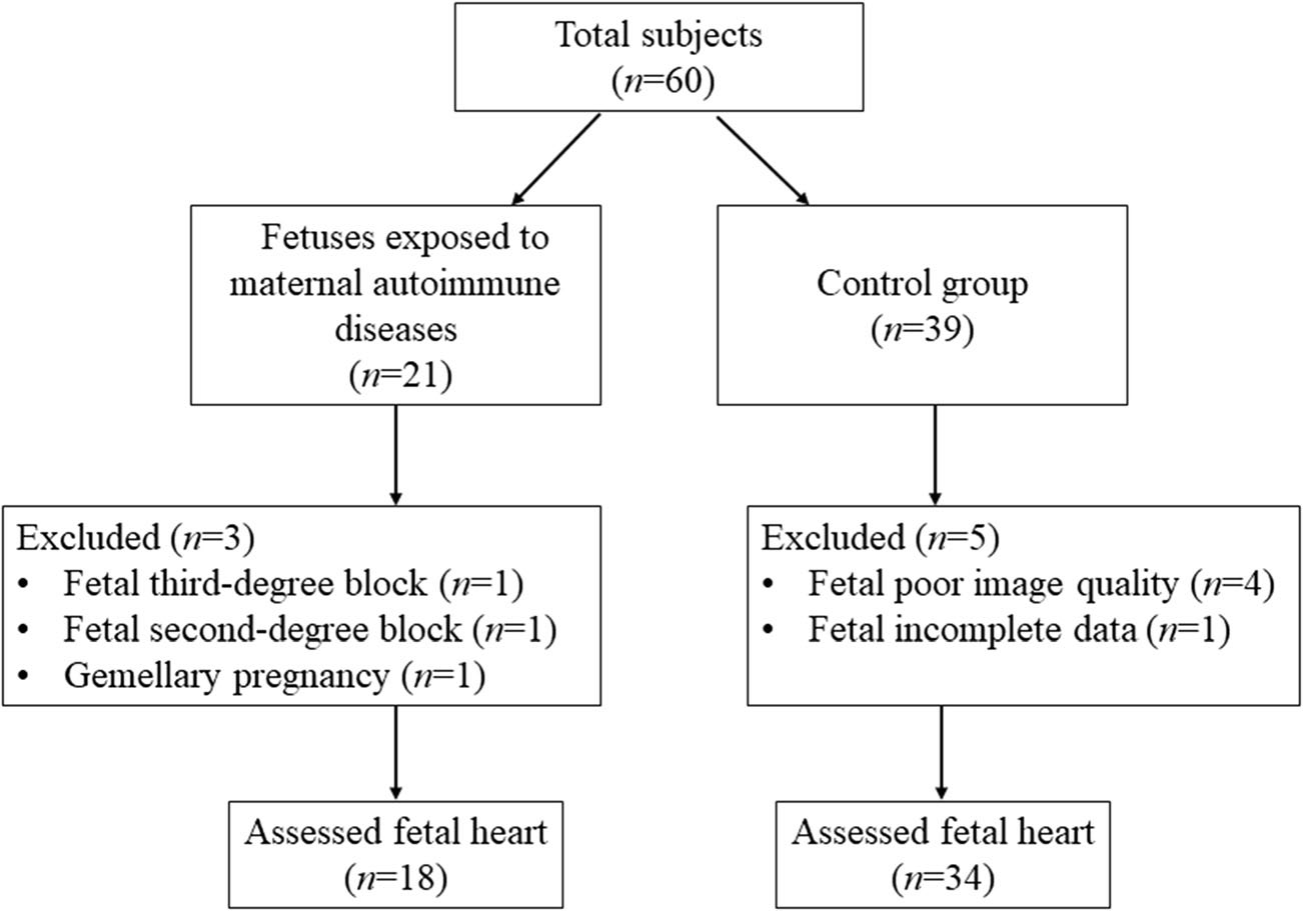
Flowchart of considered participants for study

A total of 52 fetuses, including those exposed to maternal AD (*n* = 18) and healthy controls (*n* = 34), were selected. Maternal AD included rheumatoid arthritis (*n* = 3, 17%), systemic lupus erythematosus (*n* = 5, 28%), undifferentiated connective tissue disease (*n* = 3, 17%), Sjogren’s syndrome (*n* = 5, 28%), and idiopathic thrombocytopenic purpura (*n* = 2, 11%). All cases were anti-SSA/Ro60 antibody positive, 17% (*n* = 3) were anti-SSB/La antibody positive, while 50% (*n* = 9) were anti-Ro52 antibody positive. The enrollment flowchart is presented in Fig. 3. The characteristics of the subjects are reported in Table 1. The maternal baseline characteristics, the gestational age during ultrasound examination, fetal weight, and fetoplacental Doppler analysis were comparable between the two groups.

**Table 1:** Baseline maternal and fetal characteristics

Parameters	Control group (*n* = 34)	Maternal AD group (*n* = 18)	*P*
Maternal characteristics			
Maternal age (y)	31 ± 4	31 ± 3	0.933^a^
Height (cm)	1.63 (1.60 to 1.66)	1.65 (1.62 to 1.68)	0.192^b^
Weight (kg)	61.75 (57.00 to 68.50)	63.50 (58.00 to 67.50)	0.765^b^
BMI (kg/m^2^)	24.17 ± 3.22	23.88 ± 2.89	0.757^a^
Systolic blood pressure (mm Hg)	110 (107 to 111)	110 (105 to 115)	0.946^b^
Diastolic blood pressure (mm Hg)	70 (60 to 75)	65 (62 to 70)	0.146^b^
Multiparity (*n*)	8 (24%)	4 (22%)	0.601^c^
History of having a baby with CHB/neonatal lupus (*n*)	0 (0%)	0 (0%)	
Anti-SSA/Ro60 antibody positive	0 (0%)	18 (100%)	
Anti-SSB antibody positive	0 (0%)	3 (17%)	0.037^d^
Anti-Ro-52 antibody positive	0 (0%)	9 (50%)	<0.001^d^
Methylprednisolone	0 (0%)	5 (28%)	0.003^d^
Hydroxychloroquine (HCQ), 100 mg bid	0 (0%)	18 (100%)	
Fetoplacental ultrasound evaluation			
Gestational age (wk)	23.16 ± 1.09	23.57 ± 1.76	0.398^a^
Estimated fetal weight (g)	506.03 ± 56.00	509.17 ± 24.44	0.822^a^
Umbilical artery PI	1.13 ± 0.15	1.08 ± 0.10	0.197^a^
Middle cerebral artery PI	1.34 (1.24 to 1.50)	1.31 (1.23 to 1.44)	0.525^b^
Cerebroplacental ratio	1.20 (1.08 to 1.38)	1.20 (1.10 to 1.35)	0.672^b^
Ductus venosus PI	0.55 ± 0.18	0.64 ± 0.15	0.077^a^

Normally distributed variables are given as mean ± standard deviation or number (percentage). Non-normally distributed variables are given as median and interquartile range. ^a^*P*-value by *t*-test. ^b^*P*-value by Mann-Whitney *U* test. ^c^*P*-value by chi-squared test. ^d^*P*-value by Fisher’s exact probability test. *AD*, autoimmune disease; *BMI*, body mass index; *CHB*, complete heart block; *PI*, pulsatility index

### Morphometric and functional echocardiographic parameters

The two groups did not differ significantly on conventional echocardiographic data for systolic and diastolic function of both ventricles, and mechanical PR interval (Table 2).

**Table 2:** Conventional fetal echocardiographic data

Parameters	Control group (*n* = 34)	Maternal AD group (*n* = 18)	*P*
Cardiothoracic ratio	28.50 ± 2.96	29.00 ± 2.49	0.544^a^
Left atrial area (cm^2^)	0.77 ± 0.15	0.83 ± 0.09	0.106^a^
Right atrial area (cm^2^)	0.74 (0.67 to 0.82)	0.78 (0.75 to 0.86)	0.090^b^
Global sphericity index	0.40 ± 0.05	0.38 ± 0.07	0.165^a^
Systolic function			
Left SF (%)	49.57 ± 7.61	50.28 ± 4.35	0.717^a^
MAPSE (mm)	4.59 ± 0.50	4.78 ± 0.57	0.216^a^
TAPSE (mm)	6.18 ± 0.57	6.37 ± 0.38	0.107^a^
Diastolic function			
Tricuspid E/A ratio	0.70 (0.60 to 0.74)	0.64 (0.59 to 0.70)	0.232^b^
Mitral E/A ratio	0.62 ± 0.05	0.66 ± 0.10	0.155^a^
Global function			
LV-MPI	0.44 ± 0.06	0.45 ± 0.08	0.838^a^
RV-MPI	0.46 ± 0.06	0.47 ± 0.06	0.760^a^
Mechanical PR interval (ms)	120 (110 to 124)	124 (119 to 128)	0.105^b^

Normally distributed variables are given as mean ± standard deviation. Non-normally distributed variables are given as median and interquartile range. ^a^*P*-value by *t*-test. ^b^*P*-value by Mann-Whitney *U* test. *AD*, autoimmune disease; *LV-MPI*, left ventricular myocardial performance index; *MAPSE*, mitral annular plane systolic excursion; *RV-MPI*, right ventricular myocardial performance index; *SF*, shortening fraction; *TAPSE*, tricuspid annular plane systolic excursion

### Deformation and dyssynchrony analyses by 2DSTE

Deformations and dyssynchrony analyses of the ventricles are reported in Table 3. There were no significant differences in the frame rate; fetal heart rate; GLS; and regional longitudinal strain of RVFW, LVFW, and IVS between the maternal AD group and control group (*P* > 0.05, respectively). However, 1C-DYS was significantly more prolonged in fetuses of the maternal AD group (19.50 [8.00 to 29.25] vs. 28.50 [13.50 to 39.25], *P* = 0.042). Nevertheless, 2C-DYS was not significantly prolonged.

**Table 3:** Analysis of strain and dyssynchrony measurements

Parameters	Control group (*n* = 34)	Maternal AD group (*n* = 18)	*P*
Frame rate (frames/s)	158 ± 16	158 ± 15	0.956^a^
Fetal heart rate (beats/min)	142 ± 5	140 ± 9	0.396^a^
Left ventricle			
Global longitudinal strain			
Systolic peak (%)	−17.55 (−15.68 to −19.60)	−17.85 (−16.82 to −18.62)	0.985^b^
Segmental longitudinal strain			
FW systolic peak (%)	−17.42 ± 2.67	−16.01 ± 3.28	0.100^a^
IVS systolic peak (%)	−16.01 ± 2.38	−15.35 ± 2.92	0.387^a^
1C-DYS (ms)	19.50 (8.00 to 29.25)	28.50 (13.50 to 39.25)	0.042^b^
Right ventricle			
Global longitudinal strain			
Systolic peak (%)	−16.95 (−15.32 to −17.85)	−17.20 (−13.35 to −17.95)	0.715^b^
Segmental longitudinal strain			
FW systolic peak (%)	−17.43 ± 4.03	−17.46 ± 3.13	0.976^a^
2C-DYS (ms)	18.00 (8.75 to 30.00)	20.50 (7.75 to 38.50)	0.862^b^

Normally distributed variables are given as mean ± standard deviation. Non-normally distributed variables are given as median and interquartile range. ^a^*P*-value by *t*-test. ^b^*P*-value by Mann-Whitney *U* test. *AD*, autoimmune disease; *1C-DYS*, one-chamber dyssynchrony; *2C-DYS*, two-chamber dyssynchrony; *FW*, free wall; *IVS*, interventricular septal

### Intra- and inter-observer variability

**Fig. 4:**
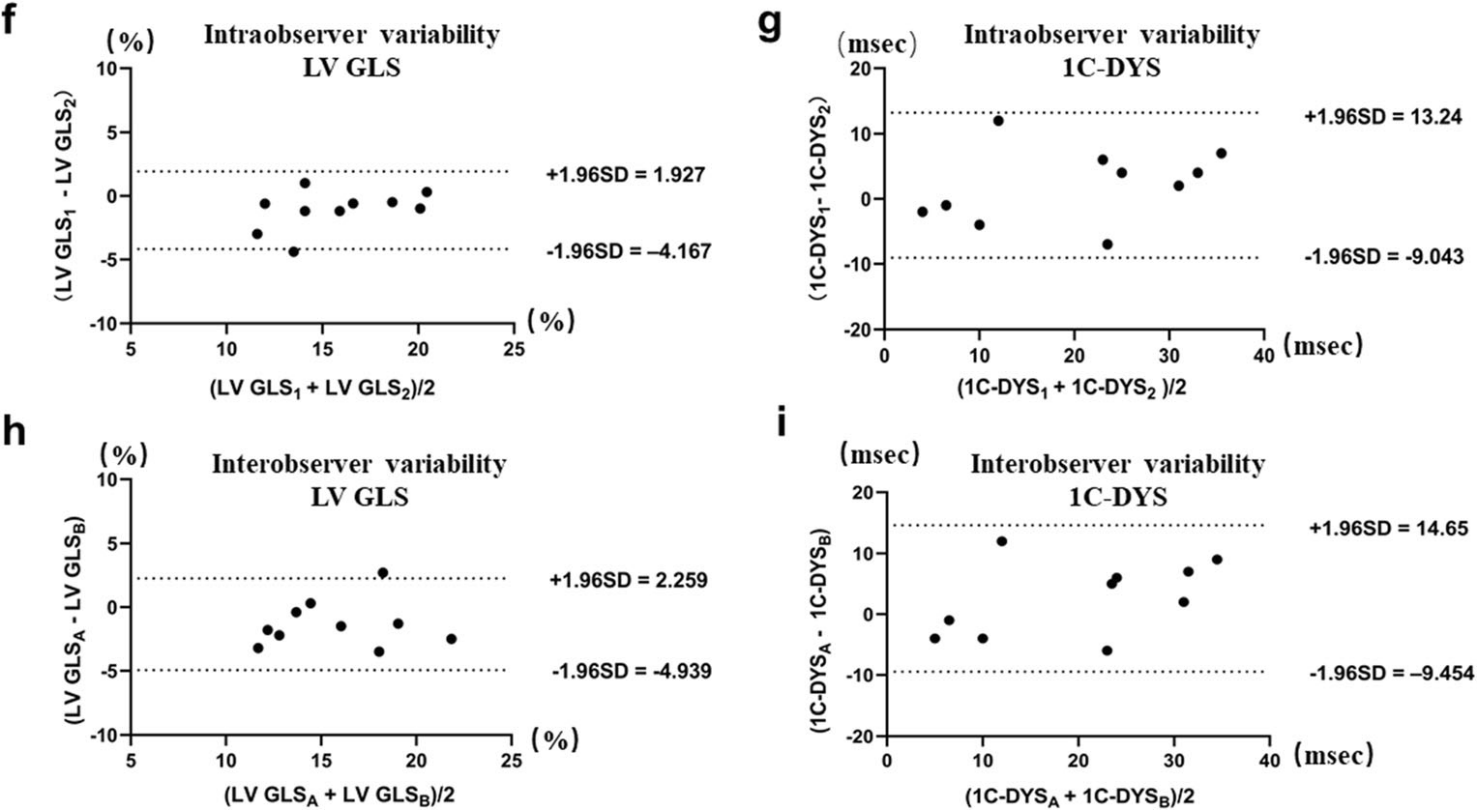
Bland-Altman plot analysis for intra-observer and inter-observer variability for LV GLS (**f**, **h**) and 1C-DYS (**g**, **i**). LV GLSA, 1C-DYSA: measures performed by the echocardiographer A. LV GLSB, 1C-DYSB: measures performed by the echocardiographer B. LV GLS1, 1C-DYS1, LV GLS2, 1C-DYS2: first and second measure performed by the same echocardiographer

The ICC for LV GLS and 1C-DYS were excellent. The ICC for the intra-observed variability were 0.849 (*P* < 0.001) for LV GLS and 0.880 (*P* < 0.001) for 1C-DYS. The ICC for the inter-observed variability were 0.815 (*P* < 0.001) for LV GLS and 0.843 (*P* < 0.001) for 1C-DYS. The Bland-Altman plots analyses for intra- and inter-observer variability are shown in Fig. 4.

## Discussion

We found that the LV and RV systolic and diastolic function remained preserved in the maternal AD group. However, LV systolic dyssynchrony of fetuses was more prolonged in mothers with AD than in healthy mothers.

Fetus of women with anti-SSA/Ro or/and anti-SSB /La antibodies have an approximate 2% risk of CHB [17, 19], and the risk approaches 19% if the woman has a history of a baby with neonatal lupus [20, 21].

Maternal antibodies can transfer to the fetus as early as 11 weeks of gestation, and these are primarily responsible for the damage to conduction tissues in the fetal heart [20]. Histologically, the heart is characterized with fibrosis, inflammation, and calcification of the atrioventricular node, resulting in blockage of signal conduction [22, 23].

Sometimes asymptomatic pregnant women with autoantibodies can have fetuses with CHB, and the bradycardia is usually noticed during a routine prenatal examination [24]. However, it is too late when the fetus has CHB because third-degree CHB cannot be reversed by drugs [25]. Strain and dyssynchrony parameters measured using 2DSTE have been used to quantify subclinical abnormalities in other fetal diseases, with a reportedly high sensitivity [8, 10]. The strain indicates myocardium deformation. GLS is defined as the average of peak systolic strain values of the ventricle segmental myocardium. The linear strain is calculated by Lagrangian formula: *S* = Δ*L*/*L*0 = (*L* − *L*0)/*L*0. Where *S* = strain, *L*0 = baseline length, and *L* = instantaneous lengths at the time of measurement. Strain was used to assess cardiac systolic function. Time to peak longitudinal strain was defined as the time from the beginning of the QRS complex on ECG to the negative peak of the longitudinal strain curve during the cardiac cycle. It was used for the quantification of mechanical dyssynchrony of segments myocardium.

To the best of our knowledge, the present study is the first to comprehensively analyze the cardiac function and systolic dyssynchrony using 2DSTE in fetuses in maternal AD.

Our data suggest that LV and RV function, as measured by conventional echocardiography and 2DSTE, were no significantly different between the fetuses of the two groups. In addition, no significant differences were found between the two groups when comparing the longitudinal strain of the LVFW, IVS, and RVFW. These results demonstrated that both the left and right systolic and diastolic ventricular values were preserved, which differs from our hypothesis. This might be explained by the fact that all mothers of the AD group were treated regularly with HCQ before and during pregnancy, and some also treated with methylprednisolone. HCQ has been suggested as a potential preventive medicine against autoimmune CHB by inhibiting Toll-like receptor signaling [26]. A study revealed that in pregnant woman with SLE and anti-SSA/Ro or anti-SSB/La antibodies, exposure to HCQ may reduce the risk of Ro-mediated fetal cardiac injury, suggesting cardioprotective effects of HCQ [27]. Prospective studies to confirm the protective effect of HCQ are required.

At present, pulsed Doppler study is used to measure the mechanical PR interval of the fetal heart [5, 17]. For fetuses of women with AD antibodies, serial measurement at 1–2-week intervals starting from 16 weeks and continuing to 28 weeks of gestation is recommended as the potential benefits surpass the risks [28]. However, a previous study showed that prolongation of mechanical PR interval was unusual and did not precede more severe blocks. Advanced block and severe cardiomyopathy can occur within 1 week of normal heart rate without initial low-degree block [17]. Thus, the role of mechanical PR interval in maternal AD needs to be studied further. In our study, the mechanical PR interval was not significantly prolonged in the maternal AD group than in the control group.

Ventricular systolic dyssynchrony has an important impact on ventricular function and structure [29]. Our study shows that the 1C-DYS was significantly more prolonged in fetuses of women with maternal AD and positive antibodies, whereas 2C-DYS was not significantly different. These results indicate the damage to the left ventricular conduction system of fetuses. The reason for this phenomenon might be the anti-SSA/Ro or/and anti-SSB /La antibodies, which damage the conduction tissues of the fetal heart. Nevertheless, these findings should be confirmed in larger controlled trials. Moreover, further investigations are required to assess mechanical dyssynchrony as a predictor of poor outcome and as a monitoring tool in this population.

The present study has several limitations. First, this was a single-center study and the sample size was small. Second, the serum antibody was detected by the EUROLINE ANA Profile (IgG) (EUROIMMUN medical diagnosis Co., Ltd), which is a qualitative diagnosis assay and could not measure the anti-Ro/La titer. Hence, we should analyze the association between anti-Ro “level” and echocardiographic parameters in a future study. Third, on account of the small size of the fetal heart, the circumferential and radial strain parameters were not analyzed. The small size of fetal heart might have also led to reduced accuracy and over smoothing due to suboptimal spatial resolution. Fourth, due to infeasible fetal electrocardiograph and lack of knowledge on perfect time of AV closure, only the time to peak strain could be measured. Thus, the exact systolic strain values and time to peak systolic strain might have been overestimated. Finally, this was a cross-sectional study, and regular follow-up should be conducted in future studies.

Despite these limitations, the study demonstrated that LV longitudinal strain and mechanical dyssynchrony parameter derived by 2DSTE measurement showed good feasibility and reproducibility, as reported in previous studies [6, 7].

In conclusion, a comprehensive evaluation of fetal cardiac function and dyssynchrony is feasible in maternal AD by 2DSTE. Fetuses exposed to maternal AD with autoimmune antibodies (anti-SSA/Ro or/and anti-SSB /La) showed increased levels of left ventricular dyssynchrony in comparison to fetuses of healthy pregnant women. The 2DSTE could provide additional information over conventional fetal echocardiography in detecting subclinical damage to the left ventricle conduction system of fetal heart in maternal AD, which may help clinicians better understand and manage those pregnant women and their fetuses.

## Data Availability

Not applicable.

## References

[1] Tincani A, Rebaioli CB, Frassi M, Taglietti M, Gorla R, Cavazzana I, Faden D, Taddei F, Lojacono A, Motta M, Trepidi L, Meroni P, Cimaz R, Ghirardello A, Doria A, Pisoni MP, Muscara M, Brucato A, Pregnancy Study Group of Italian Society of R (2005). Pregnancy and autoimmunity: maternal treatment and maternal disease influence on pregnancy outcome. Autoimmun Rev.

[2] Eftekhari P, S L, Lezoualc’h F, Mialet J, Gastineau M, Briand J-P, Isenberg DA, F GJ, Argibay J, Fischmeister R, Muller S, Hoebeke J (2000). Anti-SSA/Ro52 autoantibodies blocking the cardiac 5-HT4 serotoninergic receptor could explain neonatal lupus congenital heart block. Eur J Immunol.

[3] Buyon JP, Hiebert R, Copel J, Craft J, Friedman D, Katholi M, Provost TT, Reichlin M, Rider L, Rupel A, Susan Saleeb IW, Skovron ML (1998). Autoimmune-associated congenital heart block: demographics,mortality, morbidity and recurrence rates obtained from a National Neonatal Lupus Registry. Pediatr Cardiol.

[4] Canobbio MM, Warnes CA, Aboulhosn J, Connolly HM, Khanna A, Koos BJ, Mital S, Rose C, Silversides C, Stout K, American Heart Association Council on C, Stroke N, Council on Clinical C, Council on Cardiovascular Disease in the Y, Council on Functional G, Translational B, Council on Quality of C, Outcomes R (2017). Management of pregnancy in patients with complex congenital heart disease: a scientific statement for healthcare professionals from the American Heart Association. Circulation.

[5] Nii M, Hamilton RM, Fenwick L, Kingdom JC, Roman KS, Jaeggi ET (2006). Assessment of fetal atrioventricular time intervals by tissue Doppler and pulse Doppler echocardiography: normal values and correlation with fetal electrocardiography. Heart.

[6] Miranda JO, Cerqueira RJ, Ramalho C, Areias JC, Henriques-Coelho T (2018). Fetal cardiac function in maternal diabetes: a conventional and speckle-tracking echocardiographic study. J Am Soc Echocardiogr.

[7] Kapusta L, Mainzer G, Weiner Z, Deutsch L, Khoury A, Haddad S, Lorber A (2012). Second trimester ultrasound: reference values for two-dimensional speckle tracking-derived longitudinal strain, strain rate and time to peak deformation of the fetal heart. J Am Soc Echocardiogr.

[8] Krause K, Mollers M, Hammer K, Falkenberg MK, Mollmann U, Gorlich D, Klockenbusch W, Schmitz R (2017). Quantification of mechanical dyssynchrony in growth restricted fetuses and normal controls using speckle tracking echocardiography (STE). J Perinat Med.

[9] Storsten P, Aalen JM, Boe E, Remme EW, Gjesdal O, Larsen CK, Andersen OS, Eriksen M, Kongsgaard E, Duchenne J, Voigt JU, Smiseth OA, Skulstad H (2020). Mechanical effects on right ventricular function from left bundle branch block and cardiac resynchronization therapy. JACC Cardiovasc Imaging.

[10] Drop M-CV, Möllers M, Hammer K, Oelmeier de Murcia K, Falkenberg MK, Braun J, Eveslage M, Köster HA, Klockenbusch W, Steinhard J, Schmitz R (2019). Strain and dyssynchrony in fetuses with congenital heart disease compared to normal controls using speckle tracking echocardiography (STE). J Perinat Med.

[11] Guan Z, Liu S, Wang Y, Meng P, Zheng X, Jia D, Yang J, Ma C (2019). Left ventricular systolic dysfunction potentially contributes to the symptoms in heart failure with preserved ejection fraction. Echocardiography.

[12] Carvalho JS, Allan LD, Chaoui R, Copel JA, GR DV, Hecher K, Lee W, Munoz H, Paladini D, Tutschek B, Yagel S, International Society of Ultrasound in O, Gynecology (2013). ISUOG Practice Guidelines (updated): sonographic screening examination of the fetal heart. Ultrasound Obstet Gynecol.

[13] Salomon LJ, Alfirevic Z, Da Silva CF, Deter RL, Figueras F, Ghi T, Glanc P, Khalil A, Lee W, Napolitano R, Papageorghiou A, Sotiriadis A, Stirnemann J, Toi A, Yeo G (2019). ISUOG Practice Guidelines: ultrasound assessment of fetal biometry and growth. Ultrasound Obstet Gynecol.

[14] Hadlock FP, Harrist RB, Carpenter RJ, Deter RL, Park SK (1984). Sonographic estimation of fetal weight. Radiology.

[15] Practice Guidelines ISUOG (2013). Use of Doppler ultrasonography in obstetrics. Ultrasound Obstet Gynecol.

[16] Miranda JO, Ramalho C, Henriques-Coelho T, Areias JC (2017). Fetal programming as a predictor of adult health or disease: the need to reevaluate fetal heart function. Heart Fail Rev.

[17] Friedman DM, Kim MY, Copel JA, Davis C, Phoon CK, Glickstein JS, Buyon JP, Investigators P (2008). Utility of cardiac monitoring in fetuses at risk for congenital heart block: the PR interval and dexamethasone evaluation (PRIDE) prospective study. Circulation.

[18] Voigt JU, Pedrizzetti G, Lysyansky P, Marwick TH, Houle H, Baumann R, Pedri S, Ito Y, Abe Y, Metz S, Song JH, Hamilton J, Sengupta PP, Kolias TJ, d’Hooge J, Aurigemma GP, Thomas JD, Badano LP (2015). Definitions for a common standard for 2D speckle tracking echocardiography: consensus document of the EACVI/ASE/industry task force to standardize deformation imaging. Eur Heart J Cardiovasc Imaging.

[19] Brucato A, Frassi M, Franceschini F, Cimaz R, Faden D, Pisoni MP, Muscara M, Vignati G, Stramba-Badiale M, Catelli L, Lojacono A, Cavazzana I, Ghirardello A, Vescovi F, Gambari PF, Doria A, Meroni PL, Tincani AA (2001). Risk of congenital complete heart block in newborns of mothers with anti-Ro/SSA antibodies detected by counterimmunoelectrophoresis. Arthritis Rheum.

[20] Izmirly PM, Saxena A, Kim MY, Wang D, Sahl SK, Llanos C, Friedman D, Buyon JP (2011). Maternal and fetal factors associated with mortality and morbidity in a multi-racial/ethnic registry of anti-SSA/Ro-associated cardiac neonatal lupus. Circulation.

[21] Llanos C, Izmirly PM, Katholi M, Clancy RM, Friedman DM, Kim MY, Buyon JP (2009). Recurrence rates of cardiac manifestations associated with neonatal lupus and maternal/fetal risk factors. Arthritis Rheum.

[22] Llanos C, Friedman DM, Saxena A, Izmirly PM, Tseng CE, Dische R, Abellar RG, Halushka M, Clancy RM, Buyon JP (2012). Anatomical and pathological findings in hearts from fetuses and infants with cardiac manifestations of neonatal lupus. Rheumatology (Oxford).

[23] Ho SY, Esscher E, Anderson RH, Michaksson M (1986). Anatomyof congenitalcompleteheart block and relationto maternal anti-Ro antibodies. Am J Cardiol.

[24] Lazzerini PE, Capecchi PL, Laghi-Pasini F, Boutjdir M (2017). Autoimmune channelopathies as a novel mechanism in cardiac arrhythmias. Nat Rev Cardiol.

[25] Panaitescu AM, Nicolaides K (2018). Maternal autoimmune disorders and fetal defects. J Matern Fetal Neonatal Med.

[26] Wainwright B, Bhan R, Trad C, Cohen R, Saxena A, Buyon J, Izmirly P (2020). Autoimmune-mediated congenital heart block. Best Pract Res Clin Obstet Gynaecol.

[27] Izmirly PM, Kim MY, Llanos C, Le PU, Guerra MM, Askanase AD, Salmon JE, Buyon JP (2010). Evaluation of the risk of anti-SSA/Ro-SSB/La antibody-associated cardiac manifestations of neonatal lupus in fetuses of mothers with systemic lupus erythematosus exposed to hydroxychloroquine. Ann Rheum Dis.

[28] Donofrio MT, Moon-Grady AJ, Hornberger LK, Copel JA, Sklansky MS, Abuhamad A, Cuneo BF, Huhta JC, Jonas RA, Krishnan A, Lacey S, Lee W, Michelfelder EC, Rempel GR, Silverman NH, Spray TL, Strasburger JF, Tworetzky W, Rychik J, American Heart Association Adults With Congenital Heart Disease Joint Committee of the Council on Cardiovascular Disease in the Y, Council on Clinical Cardiology CoCS, Anesthesia, Council on C, Stroke N (2014). Diagnosis and treatment of fetal cardiac disease: a scientific statement from the American Heart Association. Circulation.

[29] Yang Y, Liu C, Tian J, Ding X, Yu S, Bian S, Yang J, Qin Z, Zhang J, Ke J, Yuan F, Zhang C, Rao R, Huang L (2020). Preliminary study of right ventricular dyssynchrony under high-altitude exposure: determinants and impacts. Front Physiol.

